# COVID-19 Outbreak: A Call to Arms for the World Healthcare Systems

**DOI:** 10.3390/ijerph20247175

**Published:** 2023-12-13

**Authors:** Vincenzo Russo, Francesco Saverio Mennini

**Affiliations:** 1 Department of Medical Translational Sciences, University of Campania “Luigi Vanvitelli”—Monaldi Hospital, 80131 Naples, Italy; 2 Centre for Economics and International Studies—Economic Evaluation and Health Technology Assessment, Faculty of Economics, University of Rome “Tor Vergata”, 00133 Rome, Italy; f.mennini@uniroma2.it

Coronavirus Disease 2019 (COVID-19) outbreak has placed a strong pressure on worldwide healthcare systems over the last years, testing their capacity to withstand stress. During the first wave, most infected patients were managed in an outpatient setting, until the clinical course of the disease was complicated by the onset of severe interstitial pneumonia and respiratory distress syndrome that required hospital admission [1]. The household transmission of Severe Acute Respiratory Syndrome CoronaVirus 2 (SARS-CoV2) and the outcome of its infection were correlated with several socio-economic variables, including the economic inequality of the geographical area [2]. The national healthcare systems had to rapidly re-organize the diagnostic and therapeutic pathways, reallocating health resources and hospital beds, to manage COVID-19 patients. Moreover, some governments, such as Italy, adopted strict rules characterized by national lockdowns, partial nationwide movement restriction, mandatory mask use, and social distancing in an attempt to contain the virus. Consequently, some changes in the pattern of hospital activities or admissions for diseases other than COVID-19 were observed, such as drastic reduction in the hospitalizations and interventional procedures for cardiovascular diseases, both elective and urgent [3]. We cannot exclude the hypothesis that the delays in the management of acute cardiovascular disease with severe prognostic impact may have increased the non-COVID-19 out-of-hospital mortality in several countries [4]. However, an indirect positive effect of the emergency response to the COVID-19 outbreak was the remarkable increase in the use of telemedicine for the follow-up visits of patients with non-COVID-19-related chronic disease, such as remote monitoring for the follow-up of CIEDs recipients [5,6]. Telehealth helped us to provide continuous care, reducing disease exposure for staff and physicians. It represents a useful tool that will continue to be used in future. In this scenario, a nurse-based care delivery model involving teleconsultation may be a simple and well-tolerated strategy that ensures the continuity of care and outpatient management for patients with cardiovascular diseases during the COVID-19 pandemic [7,8,9]. The lack of adequate reimbursement and the absence of sharing standards for medical teleconsultation represent the most important barriers to the implementation of telehealth tools in clinical practice [10]. The present Special Issue summarized some experiences of preventive and social medical activities during and after the COVID-19 outbreak, with particular reference to Italy, the first Western country to be hit by SARS-CoV2.

## References

[1] De Koning E.R., Boogers M.J., Beeres S.L., Kramer I.D., Dannenberg W.J., Schalij M.J. (2022). Managing Hospital Capacity: Achievements and Lessons from the COVID-19 Pandemic. Prehosp Disaster Med..

[2] Italy: Government of Italy Decree of the President of the Council of Ministers. https://www.gazzettaufficiale.it/eli/id/2020/03/09/20A01558/sg.

[3] Caso V.M., Sperlongano S., Liccardo B., Romeo E., Padula S., Arenga F., D’Andrea A., Caso P., Golino P., Nigro G. (2022). The Impact of the COVID-19 Outbreak on Patients’ Adherence to PCSK9 Inhibitors Therapy. J. Clin. Med..

[4] De Rosa S., Spaccarotella C., Basso C., Calabro M.P., Curcio A., Filardi P.P., Mancone M., Mercuro G., Muscoli S., Nodari S. (2020). Reduction of hospitalizations for myocardial infarction in Italy in the COVID-19 era. Eur. Heart J..

[5] Pronovost P.J., Cole M.D., Hughes R.M. (2022). Remote Patient Monitoring During COVID-19: An Unexpected Patient Safety Benefit. JAMA.

[6] De Larochellière H., Champagne J., Sarrazin J.F., Steinberg C., Philippon F., Roy K., Molin F., O’Hara G., Plourde B., Blier L. (2020). Findings of remote monitoring of implantable cardioverter defibrillators during the COVID-19 pandemic. Pacing Clin. Electrophysiol..

[7] Grata-Borkowska U., Sobieski M., Drobnik J., Fabich E., Bujnowska-Fedak M.M. (2022). Perception and Attitude toward Teleconsultations among Different Healthcare Professionals in the Era of the COVID-19 Pandemic. Int. J. Environ. Res. Public Health.

[8] Mohan B., Singh B., Singh K., Naik N., Roy A., Goyal A., Singh G., Aggarwal S., Saini A., Tandon R. (2022). Impact of a nurse-led teleconsultation strategy for cardiovascular disease management during COVID-19 pandemic in India: A pyramid model feasibility study. BMJ Open.

[9] Yi X., Jamil N.B., Gaik I.T.C., Fee L.S. (2020). Community nursing services during the COVID-19 pandemic: The Singapore experience. Br. J. Community Nurs..

[10] Carrillo de Albornoz S., Sia K.L., Harris A. (2022). The effectiveness of teleconsultations in primary care: Systematic review. Fam. Pract..

